# Experimental infection of grey partridges with Bagaza virus: pathogenicity evaluation and potential role as a competent host

**DOI:** 10.1186/s13567-018-0536-8

**Published:** 2018-05-09

**Authors:** Cristina Cano-Gómez, Francisco Llorente, Elisa Pérez-Ramírez, Ramón C. Soriguer, Mathieu Sarasa, Miguel Ángel Jiménez-Clavero

**Affiliations:** 1 Centro de Investigación en Sanidad Animal (INIA-CISA), Ctra. Algete a El Casar, 28130 Valdeolmos, Spain; 2 Estación Biológica de Doñana (EBD-CSIC), Américo Vespucio, s/n, 41092 Seville, Spain; 3 Fédération Nationale des Chasseurs (FNC), 13 rue du Général Leclerc, 92136 Issy-les-Moulineaux cedex, France; 4 BEOPS, 1 Esplanade Compans Caffarelli, 31000 Toulouse, France; 5 CIBER Epidemiología y Salud Pública (CIBERESP), Madrid, Spain

## Abstract

Bagaza virus (BAGV; synonymous to Israel turkey meningoencephalomyelitis virus, ITV) is a relevant arthropod-borne epornitic flavivirus. In its first emergence in Europe (southern Spain, 2010) BAGV caused an outbreak, severely affecting red-legged partridges and common pheasants. The effects (pathogenicity, role as reservoir host) of BAGV in other European phasianids are unknown. To fill this gap, grey partridges were experimentally infected with BAGV. The clinical course of the disease was severe, with neurological signs, significant weight loss and 40% mortality. Low viral loads in the blood and the absence of contact transmission suggest a limited—if any—role on BAGV transmission for this European phasianid.

## Introduction, methods and results

Bagaza virus (BAGV), synonymous to Israel turkey meningoencephalomyelitis virus (ITV), is a mosquito-borne epornitic virus belonging to the Ntaya serocomplex within the *Flavivirus* genus of the family *Flaviviridae*. It causes a severe neurological disease in poultry and wild bird species [1, 2]. The first description of ITV was reported in 1959 from an outbreak in turkeys in Israel [3]. Seven years later, BAGV was isolated from a mosquito pool in the locality of Bagaza (Central African Republic). Subsequently, full genome sequence comparisons confirmed that both viruses are the same species [1, 4] and a new name for these viruses, “Avian meningoencephalomyelitis virus” (Acronym: AMEV), was proposed. Until recently, BAGV/ITV was restricted to sub-Saharan Africa (Central African Republic, Mauritania, Senegal and South Africa), Israel and India [5–8], but in 2010 it emerged for the first time in Europe causing an outbreak that affected red-legged partridges (*Alectoris rufa*) and common pheasants (*Phasianus colchicus*) in southern Spain [9]. Specific neutralizing antibodies were detected in the same area one year later in juvenile red-legged partridges, suggesting virus overwintering [10].

The emergence of BAGV in Spain in 2010 caused high mortality rates, in particular in red-legged partridges, demonstrating high virulence and spread ability in game birds and increasing the perception of risk associated with its introduction in Europe [9]. A study in game birds naturally infected in this outbreak showed differences in pathogenicity for red-legged partridges, ring-necked pheasants and common wood pigeons [11]. In addition, experimentally infected red-legged partridges showed high susceptibility to BAGV infection and the disease was spread by direct contact [12].

The grey partridge (*Perdix perdix*) is one of most widespread and popular game birds in Europe and western Asia. In Europe, in the last half-century the species has suffered an important demographic decline mainly due to the loss and degradation of breeding habitats and the related implications on demographic traits [13–16]. In many countries this species is bred in captivity and released into the wild for hunting and restocking purposes [14, 15, 17].

The grey partridge has an important ecological and economic value as a game bird in Europe. For this reason, the conservation concerns cited above and the pathogenic effects previously observed in BAGV-infected red-legged partridges, we studied to what extent BAGV might affect the grey partridge in terms of mortality and morbidity, as well as to define its potential role as a natural reservoir.

Thirty-six five-month-old grey partridges (*Perdix perdix*) were obtained from the Gibovendee breeding facility (Chambretaud, France). Previous exposure to WNV, USUV and BAGV was determined by analyzing specific antibodies by VNT and viral RNA in feathers using real-time RT-PCR [18, 19]. The grey partridges were distributed in homogenous experimental groups composed of 50% males and 50% females and housed in three separate cages in our BSL-3 animal facilities. An “inoculated group” (*n* = 10) and a “necropsy group” (*n* = 12), housed in two cages, received a subcutaneous inoculation in the neck of approximately 2 × 10^5^ pfu/individual of BAGV (strain Spain RLP Hcc2/2010, *GenBank*: KR108245) diluted in PBS with 0.2% BSA, as previously described [12]. A third group, “in-contact group”, composed of 4 non-inoculated grey partridges, was kept together with the “inoculated group”. Finally, a “control group” (*n* = 10), housed in a third cage, was sham-inoculated with an equivalent volume of PBS with 0.2% BSA and handled as the infected group. All cages were placed in the same room.

After infection, the birds were observed daily to monitor clinical condition and weight for up to 18 dpi. In the inoculated and control groups, sampling of blood, immature rump feathers and oropharyngeal and cloacal swabs was carried out at 1, 3, 5, 7, 9, 11 and 15 dpi while at 18 dpi only feathers and blood were collected. As for the in-contact group, the same samples were collected at 5, 7, 9, 11, 15 and 18 dpi, following the previously described procedure [12]. Birds in the necropsy group were humanely euthanized by intravenous injection of embutramide (T61 ^®^, Intervet—Schering-Plough, Madrid, Spain) at 3, 4, 7 and 11 dpi (3 birds each day). Similarly, severely affected birds, showing irreversible clinical signs were euthanized in the same way for bioethical reasons. At the end of the experiment (18 dpi), survivors from the inoculated and contact groups were also euthanized as well as the control group. All birds were subjected to complete post-mortem examination and tissue samples from the brain, heart, kidney, spleen and liver were collected to evaluate viral tissue tropism by real-time RT-PCR. Samples yielding Ct values > 40 were considered negative.

The analysis of viral RNA loads in the blood, swabs, feathers and organs, and of specific antibodies in the serum, was performed as previously described [12]. Also, BAGV viremia levels were measured by a standard plaque formation assay [20]. Differences in survival curves of infected and control groups were calculated by the Kaplan–Meier method and analyzed by a log-rank test using SPSS15. Differences between groups in body weight along the experiment were analyzed using a general linear mixed model. Treatment (control or infected) and days post-infection (dpi) were entered as fixed factors whereas individual identity was included as a random term in the model. This analysis was run in R 3.4.1 (R Core Team 2017).

**Figure 1:**
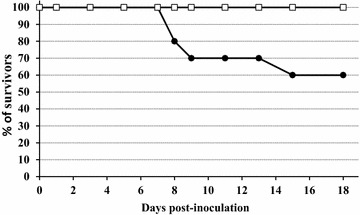
**Survival rate of grey partridges after inoculation of BAGV.** Closed circles represent the BAGV-inoculated group. Open squares represent the control group.

Most inoculated partridges developed a progressive neurological disorder showing weakness, apathy, unresponsiveness, ruffled feathers, ataxia and prostration. Some of them suffered severe neurological symptoms like partial or complete paralysis as well as ocular lesions. Four of them, showing irreversible clinical signs, were euthanized for bioethical reasons, yielding a mortality rate of 40%. These lethal cases occurred at 8 dpi (*n* = 2), 9 dpi (*n* = 1) and 15 dpi (*n* = 1) (Figure 1). The differences in survival curves between control and infected groups were statistically significant (*p* = 0.029).

**Figure 2:**
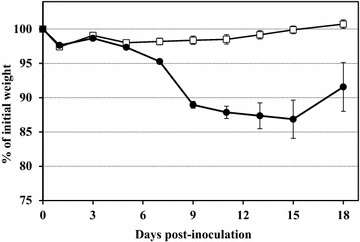
**Weight curves of BAGV infected and control groups throughout the experiment.** Closed circles represent the BAGV-inoculated group, while open squares represent the control group. Error bars represent the standard deviation of the data.

BAGV infection strongly affected the body weight of the infected group as compared to the control group throughout the experiment (time × treatment interaction: F_(1,165.9)_ = 117.8, *p* < 0.001). The weight loss started at 7 dpi and reached a maximum level at 15 dpi (13% loss of initial weight). At 18 dpi a slight recovery of body weight was observed in the survivors of the infected group but the average weight was still much lower than that of the control group (Figure 2).

**Figure 3:**
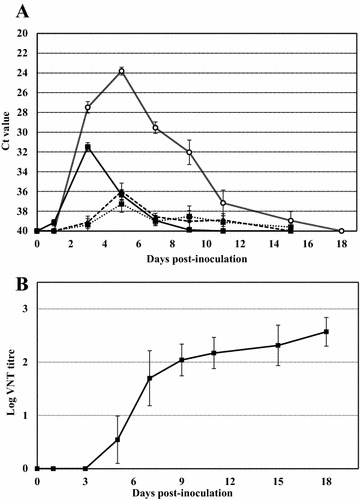
**Viral RNA load and immune response in BAGV-inoculated grey partridges. A** Viral genome load in blood, swabs (oropharyngeal, cloacal) and immature feathers. The course of the viral RNA load in each type of sample, measured by real-time RT-PCR, is represented at different dpi. The discontinuous line indicates viral RNA load in oral swabs; the dotted line the viral RNA load in cloacal swabs; the double line and open circles the viral RNA load in feathers; the continuous line the viral RNA load in blood. Error bars represent the standard deviation of the data. **B** Antibody response to BAGV in serum. Mean log titres of BAGV-NtAb (detected by VNT) measured on different dpi. Error bars represent the standard deviation of the data.

Viral RNA in the blood was detectable in the inoculated individuals from day 1 to day 7 post-infection (at 9 dpi it was detected only in one bird very close to detection limits). Peak viral RNA load in the blood was observed in all infected birds at 3 dpi (Figure 3A). Viremia, as measured by standard plaque-formation, was only detected at 3 dpi with a mean titer of 2.29 × 10^4^ pfu/mL. Infected partridges developed specific NtAb whereas there was no serocoversion of either in-contact or control birds. First NtAb were detected at 5 dpi and by day 7 post-infection all infected partridges had developed high NtAb titers (ranging from 1:160 to 1:640) that persisted until the end of the experiment (Figure 3B). Viral shedding through the oropharyngeal and cloacal routes reached a peak at 5 dpi. However, the viral RNA load was low in oropharyngeal and cloacal swabs throughout the experiment (Figure 3A). In contrast, the viral RNA load in immature feathers was present at much higher concentrations and could be detected up to 15 dpi (Figure 3A).

BAGV inoculation in grey partridges led to a systemic infection since the viral RNA was detected in all organs analyzed at different times post-infection (Table 1). The highest viral RNA loads were found in the heart, kidney, spleen and liver at 4 dpi, only 1 day after the viremia peak. However, the highest viral RNA load in the brain was observed later, at 7 dpi. At 11 dpi, the viral RNA load in all the organs was much lower than that found in birds euthanized at 3, 4 and 7 dpi. Those partridges that were euthanized for bioethical reasons (due to severe neurological symptoms) at 8–9 dpi showed very high viral RNA loads specifically in the heart, kidney and brain, while the partridge euthanized at 15 dpi had less viral RNA load. By the end of the experiment (at 18 dpi), the viral RNA load had decreased significantly in all the organs (Table 1).

**Table 1:** **BAGV detection by real-time RT-PCR in organs from inoculated grey partridges**

dpi	n	Viral RNA load (ct)				
		Heart	Spleen	Kidney	Brain	Liver
Inoculated group						
8^a^	2	27.1	35.3	24.5	28.4	38.0
9^a^	1	26.5	34.8	28.5	26.0	39.6
15^a^	1	> 40	38.1	35.4	32.4	> 40
18	6	39.8	37.5	33.2	37.0	39.7
Control group						
18	10	> 40	> 40	> 40	> 40	> 40
In-contact group						
18	4	> 40	> 40	> 40	> 40	> 40
Necropsy group						
3	3	29.4	28.2	30.7	34.2	33.1
4	3	25.0	25.4	27.2	32.5	28.1
7	3	27.0	30.4	27.0	29.1	34.7
11	3	36.4	37.2	32.0	32.1	39.9

The Ct corresponds to the average viral RNA load value for the different organs; Ct > 40 is considered as a negative value.

^a^Euthanized birds due to irreversible clinical signs.

In-contact and control groups remained healthy during the experiment. Remarkably, no evidence of BAGV infection (and hence of direct contact transmission) was observed in the in-contact group, based not only on the absence of clinical signs in this group, but also on the lack of virus in blood, feathers, swabs and organs and on the absence of seroconversion.

## Discussion

This study demonstrates that the grey partridge is susceptible to BAGV infection showing severe neurological symptoms, including partial and complete paralysis. In contrast, BAGV infection in red-legged partridges does not cause severe neurological disease, but apathy and weakness probably related to the intense weight loss suffered (up to 23% of initial weight) [12]. In grey partridges, the infection also caused 100% morbidity but the weight loss was less severe (13% of initial weight). The mortality rates were similar in both species (30% for red-legged partridges and 40% for grey partridges).

Viral RNA loads in blood, feathers and swabs were lower and less persistent in grey partridges as compared to red-legged partridges. However, the viral RNA load in feathers in both species was high and persistent, indicating the usefulness of feather sampling in active surveillance programs for early detection of BAGV infection. A lower viremia in grey partridges, as compared to red-legged partridges, suggests a lower competence for transmission to mosquitoes, and hence the low host capacity for the grey partridge with regard to BAGV transmission.

One of the main differences between both species was the capacity to transmit the virus by direct contact. While non-vectored transmission has been demonstrated in red-legged partridges, this was not the case for grey partridges. Similarly, contact transmission was also unsuccessful in experimental infection attempts in turkeys [21, 22]. Possibly, the much higher viral RNA loads detected in swabs and feathers in red-legged partridges could explain their capacity to transmit the virus through close contact (by feather picking or water/food contamination).

The highest viral RNA loads in organs were detected in the heart, spleen and kidney in the early days of infection (3–6 dpi). However, the maximum viral RNA load in the brain was found a bit later, at 7 dpi. Between 7 and 9 dpi, viral RNA loads in the kidney and heart remained high, similar to those observed in the brain. The lowest viral RNA loads were consistently found in the liver, as previously observed in turkeys [22]. The consistent humoral immune response developed from day 5 post infection probably contributed to the progressive viral clearance in blood, swabs and organs of survivors.

In summary, the present study proves the high susceptibility of grey partridges to BAGV infection, suffering 100% morbidity and 40% mortality. Moreover, in natural conditions, an outbreak of BAGV could have a greater impact on this species since the clinical effects and the poor body condition caused by the virus could increase the mortality rate due to difficulties with feeding and/or escaping from predators. However, the viremia and viral shedding data indicate that the role of grey partridges in the epidemiology of BAGV is limited due to its low or no competence for mosquito-borne and direct contact transmission of the virus in our experimental conditions. Further studies on the potential interactions between the grey partridge and other flaviviruses that already circulate in Europe might contribute to the conservation efforts on this game species.

## Abbreviations

BAGV: Bagaza virus
ITV: Israel turkey meningoencephalomyelitis virus
WNV: West Nile virus
USUV: Usutu virus
VNT: virus neutralization test
RT-PCR: reverse transcription polymerase chain reaction
BSL-3: biosafety level 3
pfu: plaque-forming unit
PBS: phosphate buffered saline
BSA: bovine serum albumin
dpi: days post-inoculation
Ct: threshold cycle value
NtAb: neutralizing antibodies
